# The performance of Nutri-Score as a tool in guiding and evaluating nutritional quality of food procurements for high schools in Norway

**DOI:** 10.29219/fnr.v69.11483

**Published:** 2025-10-23

**Authors:** Anna Amberntsson, Emilie Heide, Mari Mohn Paulsen, Siri Løvsjø Solberg, Anne Lene Løvhaug, Lene Frost Andersen, Marianne Hope Abel

**Affiliations:** 1 Department of Food Safety, Norwegian Institute of Public Health, Oslo, Norway; 2 Centre for Sustainable Diets, Norwegian Institute of Public Health, Oslo, Norway; 3 Department of Nutrition, Institute of Basic Medical Sciences, University of Oslo, Norway; 4 Matvalget, Oslo, Norway; 5 Institute for Nursing and Health Promotion, Oslo Metropolitan University, Oslo, Norway; 6 Department of Physical Health and Ageing, Norwegian Institute of Public Health, Oslo, Norway; 7 Centre for Evaluation of Public Health Measures, Norwegian Institute of Public Health, Oslo, Norway

**Keywords:** convergent validity, nutrient profiling models, Nutri-Score, high schools, food purchase

## Abstract

**Background:**

The Norwegian Directorate of Health has issued school meal guidelines to advocate for the availability of healthy food options. However, awareness and adherence among school officials and canteen staff are low. Moreover, the broadly formulated guidelines complicate evaluation and selection of appropriate foods. Nutri-Score might be a potential tool for guiding and evaluating food procurements.

**Objective:**

We aimed to 1) examine agreement between the Nutri-Score and the school meal guidelines, 2) investigate the potential of using Nutri-Score to guide food procurements through proposing food-group-specific ranges, and 3) propose realistic goals for food purchases in value (money spent) for recommended and non-recommended foods based on Nutri-Score distribution in a sample of schools.

**Design:**

A list of all foods procured in all high school canteens in Viken County, Norway, from January 2021 to June 2023 was obtained. The Nutri-Score 2023 version was calculated on all products. Based on the school meal guidelines, foods had been classified into three categories by nutrition experts: *foods recommended to be offered*, *foods recommended to be offered in limited amounts*, and *foods recommended not to be offered*. Agreement and disagreement were assessed using boxplots and cross tables. Linear regression presented as scatter plots was used to investigate relevant goals for food purchases.

**Results:**

There was overall good agreement between the Nutri-Score and the Norwegian school meal guidelines. We propose that foods with Nutri-Score A and B can be recommended, but for some food groups, such as bread toppings, Nutri-Score C and D can also be recommended. A goal for food procurements could be minimum 65% of total value spent on products with Nutri-Score A or B and maximum 15% on products with Nutri-Score E (C-E for beverages).

**Conclusion:**

Nutri-Score could be an effective, complementary tool for guiding and evaluating food purchases in alignment with school meal guidelines.

## Popular scientific summary

- Nutri-Score is a front-of-pack nutrition label for packaged foods and beverages.
- This paper investigates the potential of Nutri-Score as a tool for guiding and evaluating the nutritional quality of food procurements in Norwegian high schools.
- Most foods that were recommended to be offered by the Norwegian school meal guidelines obtained the most favorable Nutri-Score A or B.
- Nutri-Score-specific ranges are proposed to assist the procurer to make healthful food choices.

Schools play an important role in promoting healthy diets for children, and the link between good nutrition and education has long been recognized. A child who has good health and nutrition performs better in school, and a good education provides a child with a foundation for the future (1, 2). In addition, school-provided meals can contribute to reducing social inequalities (3). Establishing healthy dietary habits at a young age is crucial, as these habits are likely to persist into adulthood (4, 5). An important public health tool is to promote healthy food in schools (6). In Norway, most high schools have a canteen where food and beverages are sold, and a few schools also offer a free meal for breakfast or lunch (7).

The Norwegian Directorate of Health has issued guidelines for food offerings in schools (hereafter referred to as the school meal guidelines) (8). All high schools are strongly recommended to create a cafeteria or similar facility and provide daily offerings of healthy foods (9, 10). Furthermore, the school meal guidelines also provide recommendations regarding what kind of foods and beverages to be offered and highlight that the Nordic Keyhole label could be useful in identifying healthier foods (8). The Keyhole label is a voluntary front-of-package nutrition label, and its absence does not necessarily indicate that a product fails to meet the Keyhole criteria (11). Although the guidelines were meant to work as a guiding tool in the food procurement process, a survey has shown that the awareness of the guidelines is low among local school authorities, school leaders, teachers, and those responsible for the canteen in schools (12). In addition, because the school meal guidelines are broadly formulated, particularly in categories like bread spreads and hot meals, it can be difficult to evaluate which individual food items should be offered or not by the procurers and school canteens. Interpretation of the guidelines requires a comprehensive understanding of nutritional quality of food products. For example, it is recommended to offer breads that are high in fiber and whole grain and low in fat, sugar, and salt, but with no specifications of what is considered acceptable levels (8).

Questionnaires for estimating adherence to the school meal guidelines have been developed (13). However, the questionnaires do not provide an evaluation of the entire food offerings selection of schools, including foods not covered by the guidelines. The Viken County in Norway has implemented a ‘Strategy for health-promoting schools’ (14), including goals for adherence to the school meal guidelines (8). The goals were set that at least 70% of food purchases in value (money) should be spent on foods that are promoted in the school meal guidelines, and no more than 8% on discretionary foods. To evaluate the schools’ food procurements against the aims set by Viken County, a classification tool based on the school meal guidelines has previously been developed by Matvalget (15). Matvalget is a government-supported, multi-disciplinary organization that works to stimulate a more sustainable food system in Norway. Matvalget also offers individual guidance and support to schools, including analysis of food procurements, which serves as an indicator of how well the schools are complying with the school meal guidelines. In the classification tool, foods were categorized by nutrition experts in three categories primarily based on the specifications in the school meal guidelines: *recommended to be offered, recommended to be offered in limited amounts,* and *recommended not to be offered*.

Nutri-Score is a nutrient profiling system introduced as a front-of-pack nutrition label in seven European countries (16–18). Nutri-Score features a five-letter, color-coded logo, allowing consumers to recognize nutritional quality, with a grade of ‘A’ indicating the highest and ‘E’ the lowest. The Nutri-Score 2023 version has been shown to effectively differentiate food products by nutritional quality, also in a Norwegian context (19). However, limitations have been identified, including lack of discrimination between whole grain and refined grain products, poor scoring of some products with high-fat content (e.g. mayonnaise) (19), and weighting of specific nutrients (e.g. salt gives up to 20 unfavorable points, while saturated fat only gives up to 10) (20).

Currently, there is lack of awareness of and compliance with the school meal guidelines among procurers in Norwegian high schools. Furthermore, the school meal guidelines emphasize the overall selection of food but provide limited assistance during the purchasing process. Nutri-Score might be a potential tool for both guiding procurers to select healthier foods through recommended food-group-specific Nutri-Score ranges, and evaluating public food procurements through specific goals for the purchase value of recommended and non-recommended foods according to the school meal guidelines. The overall aim of this study was to assess the potential of the Nutri-Score as a tool for guiding and evaluating the nutritional quality of food procurements in Norwegian high schools. First, we aimed to examine the agreement between the Nutri-Score and the Matvalget’s classification tool as a proxy for the school meal guidelines. Second, we aimed to investigate the potential of using Nutri-Score to guide food procurements through proposing food-group-specific ranges. Third, we aimed to propose goals for food purchases in value (money spent) in high schools for recommended and non-recommended foods based on Nutri-Score distribution in the investigated schools.

## Methods

### The Norwegian Directorate of Health’s school meal guidelines

The school meal guidelines concerning food offerings in high schools from the Norwegian Directorate of Health are presented in **Textbox 1**.

Textbox 1 School meal guidelines from the Norwegian Directorate of Health covering what foods and beverages should be offered in high schools (8).

- The food and beverage offering should be based on the Norwegian Directorate of Health’s dietary guidelines. The Nordic Keyhole label and the Bread scale could be useful tools in identifying healthier foods.
- Vegetables and fruits/berries should be offered daily.
- Bread and cereal products that are high in fiber and whole grains and low in fat, sugar, and salt should be offered.
- The selection of spreads should be varied and always include fish spreads and vegetables.
- When serving hot meals, there should be a variety of fish, meat, and vegetarian dishes.
- Cooking oils, liquid, and soft margarine should be used instead of hard margarine and butter.
- Foods with low-salt content should be prioritized. The use of salt in cooking and on foods should be limited.
- Cold water should always be available as a thirst quencher for meals.
- Low-fat milk with ≤1% fat or less should be offered daily.
- If juice is offered, the serving sizes should not exceed 250 ml.
- Sodas, cordials, and other beverages with added sugar or non-nutritive sweeteners should not be offered.
- Pastries and other products high in sugar and/or fat should be reserved for special occasions.
- Chocolate, candy, chips, and other snacks should not be offered.

### The classification tool by Matvalget

The classification tool by Matvalget was developed to reflect the school meal guidelines and to give the schools an overall description of their food offerings (15). The tool consists of three categories: *foods recommended to be offered* (e.g. bread with >50% whole grain), *foods recommended to be offered in limited amounts* (e.g. bread with ≤50% whole grain), and *foods recommended not to be offered* (e.g. white bread). Nutrition experts at Matvalget assigned all the foods purchased at high schools in Viken County to the three categories. Foods that are not specifically mentioned by the school meal guidelines were evaluated by Matvalget considering whether or not the food item can be part of an overall healthy diet. In addition, the food items were evaluated by nutritional quality (salt, sugar, saturated fat, and whole grain content). A detailed description of Matvalget’s interpretation of the school meal guidelines by food category is provided in Supplementary Table 1.

### Data collection

A list of all food products procured in all high school canteens in Viken County from January 2021 to June 2023 was obtained from Matvalget. The list covered 8,563 food products, with associated product number, supplier, food category, and Matvalget’s classification. The product list was further processed in the following steps: cleared of product number duplicates (*n* = 457), food items not covered by the Nutri-Score, for example, herbs and spices (*n* = 500), and discontinued products (*n* = 1,437). Furthermore, we decided to only include products from the three major suppliers, TINE (*n* = 291), Bama (*n* = 538), and Servicegrossisten (*n* = 3,987), since these products covered all food categories and to make it feasible to obtain detailed product information. Products from other suppliers were excluded (*n* = 1,361). Nutritional information on the products was retrieved from assortment lists from the food producers, the pre-packed foods database Tradesolution, the Norwegian food composition table, and online product information. There were 4,790 foods and beverages included in the final dataset.

Additionally, data on total food procurements (all suppliers) in the second quarter of 2023 were obtained for 10 high schools from Viken County. The data covered product names, units, and amounts purchased per product per school. To increase the probability of having variation in the nutritional quality of food procurements between the included schools, five schools that had received nutritional guidance from Matvalget and five that had not were purposely selected for the study. In total, the schools had bought 2,282 different food products to a total value of 1.635.358 Norwegian Kroner (NOK), of which 200 food items (2.5% of the total purchase value) were not covered by the Nutri-Score.

### Calculation of the Nutri-Score 2023 version

The Nutri-Score is calculated based on three distinct algorithms: the main algorithm for general foods, the algorithm for fats, oils, nuts, and seeds, and the algorithm for beverages (Supplementary Tables 2–5). The Nutri-Score 2023 version (17) was used for the calculation of all the food items in the procurement lists. To calculate the score, we used the nutritional information on content per 100 g of energy, sugars, saturated fats, salt, protein, fiber, non-nutritive sweeteners (only for beverages), and total fat (only for fats, oils, nuts, and seeds), and we estimated proportion of fruits, vegetables, and legumes (%FVL) based on the available nutritional information (only for products with a potential of obtaining points for FVL).

### Statistical analysis

The overall agreement between the Nutri-Score and the Matvalget’s classification tool as a proxy for the school meal guidelines was assessed using boxplots. Agreement was defined as a food item being classified as *recommended to be offered* while obtaining Nutri-Score A or B, a food item classified as *recommended to be offered in limited amounts* while obtaining Nutri-Score C or D, or a food item classified as *recommended not to be offered* while obtaining Nutri-Score E. Disagreement was defined as a food item being classified as *recommended to be offered* while obtaining Nutri-Score D or E, or a food item classified as *recommended to be offered in limited amounts* or *recommended not to be offered* while obtaining Nutri-Score A or B.

To investigate the potential of using Nutri-Score to guide food procurements through food-group-specific Nutri-Score ranges, we examined the agreement between the Nutri-Score and the Matvalget’s classification tool, as a proxy for the school meal guidelines, by food category using cross tables and described disagreements (according to the above definition). When the school meal guidelines did not contain sufficiently detailed information (for example, whether dried fruits should be recommended or not), the Norwegian food-based dietary guidelines (FBDGs) were used as the reference document (21). Finally, the Nutri-Score ranges that best align with school meal guidelines were proposed to guide food-group-specific procurements. The starting point was that products with Nutri-Score A or B could be offered, and for some food groups, also products with Nutri-Score C.

When exploring goals for food purchases in value (money spent), scatter plots and linear regressions were used. The percentage of the total purchase value (in NOK) that each school spent on foods categorized as *recommended to be offered* and food *recommended not to be offered* was associated with the value spent on foods categorized as Nutri-Score A or B and E, respectively. Since the C category for beverages contains items *not recommended to be offered* by the school meal guidelines, the C and D categories for beverages were also counted as unfavorable Nutri-Scores in this analysis.

Statistical analyses were performed using Stata (StataCorp Stata Statistical Software: Release 17, College Station, TX, USA).

### Ethics

No human participants or sensitive information was involved in this study. Viken County signed a written agreement regarding the use of procurement data from the schools. The schools included in the study have been anonymized.

## Results

### Agreement between the Nutri-Score and the school meal guidelines using Matvalget’s classification tool as a proxy

**Fig. 1:**
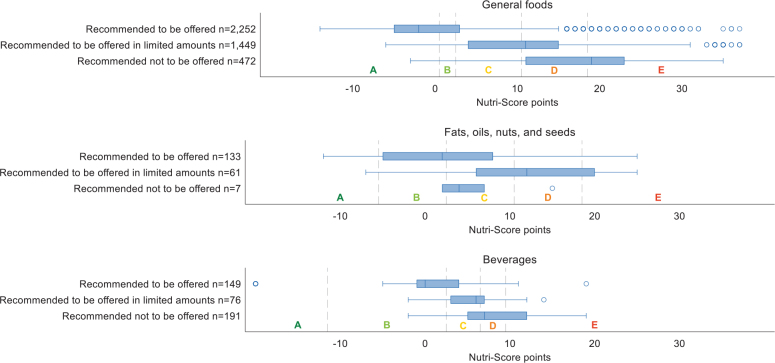
Agreement between the Nutri-Score (A to E) and the school meal guidelines, using Matvalget’s classification tool as a proxy, for foods procured in Viken County high schools (*n* = 4,790 foods). The boxplots illustrate the distribution of Nutri-Score points, which refer to the underlying algorithms for calculating the score on general foods (top), oils/fats/nuts/seeds (middle), and beverages (bottom).

Of the 4,790 included food products procured in all high school canteens in Viken County, Norway, between 2021 and 2023, 53% were categorized as products *recommended to be offered*, 33% as products *recommended to be offered in limited amounts*, and 14% as products *recommended not to be offered* according to the Matvalget’s classification tool designed to reflect the school meal guidelines (Fig. 1). The distribution of Nutri-Score A–E in the products procured was 34, 10, 21, 21, and 14%, respectively. The overall agreement between the school meal guidelines and the Nutri-Score was 67% (*n* = 3,197), while 14% (*n* = 670) of all products were in disagreement.

### Nutri-Score distribution within food groups

The food groups with the highest proportions of agreement between the Nutri-Score and the guidelines included fruit and vegetables (85%) and snacks (75%) (Table 1). The food categories with the highest proportions of disagreement were pasta and rice (68%), sauces and dressings (48%), fish products (26%), white meat (23%), grains (21%), and fats (20%). Nutritional content by Nutri-Score for the food groups is presented in Supplementary Table 6. The following paragraphs will describe in detail the agreements within food categories.

**Table 1:** Agreement between the Nutri-Score (A to E) and the school meal guidelines using the Matvalget’s classification tool as a proxy, overall and by food groups (*n* = 4,790)

	*N*	Recommended to be offered% (*N*)	Recommended to be offered in limited amounts% (*N*)	Recommended not to be offered % (*N*)
**Overall**				
A–B	2,086	71 (1,791)	**17 (271)**	**4 (24)**
C	1,006	14 (368)	30 (471)	25 (167)
D–E	1,698	**15 (375)**	53 (844)	71 (479)
**Fruits and vegetables**	1,335			
A–B	1,156	91 (1,140)	**20 (16)**	**0 (0)**
C	65	4 (54)	14 (11)	0 (0)
D–E	114	**5 (59)**	67 (54)	100 (1)
**Grains**	112			
A–B	86	86 (62)	**60 (24)**	**0 (0)**
C	18	14 (10)	20 (8)	0 (0)
D–E	8	**0 (0)**	20 (8)	0 (0)
**Bread**	297			
A–B	83	56 (59)	**13 (24)**	**0 (0)**
C	167	36 (38)	67 (126)	75 (3)
D–E	47	**8 (8)**	20 (38)	25 (1)
**Pasta and rice**	111			
A–B	99	92 (24)	**88 (75)**	**0 (0)**
C	11	8 (2)	11 (9)	0 (0)
D–E	1	**0 (0)**	1 (1)	0 (0)
**Red meat**	460			
A–B	59	83 (5)	**12 (54)**	**0 (0)**
C	91	0 (0)	20 (91)	0 (0)
D–E	310	**17 (1)**	68 (309)	0 (0)
**White meat**	152			
A–B	79	80 (47)	**35 (32)**	**0 (0)**
C	26	15 (9)	18 (17)	0 (0)
D–E	47	**5 (3)**	47 (44)	0 (0)
**Fish and seafood**	218			
A–B	122	56 (121)	**33 (1)**	**0 (0)**
C	40	18 (39)	33 (1)	0 (0)
D–E	56	**26 (55)**	33 (1)	0 (0)
**Ready meals**	313			
A–B	68	32 (51)	**13 (15)**	**5 (2)**
C	161	50 (78)	61 (69)	33 (14)
D–E	84	**18 (28)**	26 (30)	62 (26)
**Sauces and dressings**	332			
A–B	46	17 (42)	**5 (4)**	**0 (0)**
C	73	22 (57)	21 (16)	0 (0)
D–E	213	**61 (157)**	74 (56)	0 (0)
**Dairy products and eggs**	387			
A–B	68	56 (57)	**3 (9)**	**100 (2)**
C	67	20 (20)	17 (47)	0 (0)
D–E	252	**24 (25)**	80 (227)	0 (0)
**Fats**	128			
A–B	42	44 (38)	**3 (1)**	**75 (3)**
C	30	30 (26)	10 (4)	0 (0)
D–E	56	**26 (22)**	87 (33)	25 (1)
**Nuts**	73			
A–B	43	85 (40)	**13 (3)**	**0 (0)**
C	28	15 (7)	78 (18)	100 (3)
D–E	2	**0 (0)**	8 (2)	0 (0)
**Snacks**	456			
A–B	9	100 (1)	**0 (0)**	**2 (8)**
C	84	0 (0)	38 (12)	17 (72)
D–E	363	**0 (0)**	63 (9)	81 (343)
**Beverages**	416			
A–B	126	70 (104)	**17 (13)**	**5 (9)**
C	145	19 (28)	55 (42)	39 (75)
D–E	145	**11 (17)**	28 (21)	56 (107)

Bold numbers indicate foods in disagreement.

Disagreement was defined as a food item being classified as *recommended to be offered* while obtaining Nutri-Score D or E or a food item being classified as *recommended to be offered in limited amounts* or *recommended not to be offered* and obtaining Nutri-Score A or B.

Column percentages not summing up to 100 are due to rounding errors.

The school meal guidelines state that vegetables and fruits/berries should be offered daily, and it is also recommended to include vegetables as bread toppings (8)*.* In addition, foods rich in salt or sugar should be limited. According to the Norwegian FBDG, dried fruits are not included in the recommendation regarding fruits and vegetables (21). Fruits, vegetables, and legumes that were in disagreement (*n* = 75, 6%) were dried fruits, olives, fried onions, capers, and sundried tomatoes with a high-salt or high-sugar content that were *recommended to be offered* while obtaining Nutri-Score D or E, and canned fruit classified as *recommended to be offered in limited amounts* while obtaining Nutri-Score A or B (Table 1).

The school meal guidelines state that bread and cereal products that are high in fiber and whole grains and low in fat, sugar, and salt should be offered (8)*.* Bread products that were in disagreement (*n* = 32, 11%, Table 1) were categorized as *recommended to be offered* but with a Nutri-Score D or E were croutons, papadum, and gluten-free bread. Only one of the eight products contained whole grain. Furthermore, products classified as *recommended to be offered in limited amounts* while obtaining Nutri-Score A or B were hamburger buns, hot dog buns, tortillas, and gluten-free bread.

The school meal guidelines state that when serving hot meals, there should be a variety of fish, meat, and vegetarian dishes, and that foods low in salt should be prioritized. Among red meat products, 12% (*n* = 55) were in disagreement. Only one red meat product (a hamburger with added salt) was classified as *recommended to be offered* and received Nutri-Score D. Products classified as *recommended to be offered in limited amounts* while obtaining Nutri-Score A or B were lean (<10% fat), non-salted beef, lamb, and pork. In the white meat food category, products in disagreement (*n* = 35, 23%) were products of chicken with added salt, which are categorized as *recommended to be offered* but receiving Nutri-Score D, and processed products of chicken such as marinated chicken and chicken burgers were classified as *recommended to be offered in limited amounts* while obtaining Nutri-Score A or B.

According to the school meal guidelines, fish should be offered both as hot dishes and as bread toppings (8). In addition, products with a low-salt content should preferably be selected (8). All unprocessed fish and seafoods received Nutri-Score A or B while products with Nutri-Score C mostly were processed fish products (pudding and patties) and tomato-based fish spreads. Fish products in disagreement (*n* = 56, 26%; Table 1) were mostly high-salt or high-sugar products like caviar, smoked salmon, anchovy fillets, and pickled herring, which were *recommended to be offered* but received Nutri-Score D or E.

The school meal guidelines do not have specific advice for ready meals but state that in general, foods with a low content of salt should be preferred (8). Ready meals categorized as products *recommended to be offered* but obtaining Nutri-Score D or E (*n* = 28, 9%) were curry paste, soups, plant-based dinner products, mayonnaise-based salads, and crisp bread snacks, whereas products categorized as *recommended to be offered in limited amounts* or *recommended not to be offered* that got a Nutri-Score A or B (*n* = 17, 5%) were a plant-based meat substitutes, French fries, oat meal, and a powder soup (Table 1). Most ready meal products clustered in Nutri-Score C (51%).

Most sauces and dressings were categorized by Matvalget as *recommended to be offered* (77%) based on the rationale that these are normally consumed in limited amounts and can be part of an overall healthy diet. Sauces and dressings in disagreement (*n* = 157, 47%; Table 1) were barbeque sauce, sweet chili sauce, oil-based dressings, soy sauce, and mustard, which are classified as *recommended to be offered* and obtained Nutri-Score D or E. Products with Nutri-Score A or B were mostly tomato-based sauces and broths, whereas sauces and dressings with Nutri-Score C or D were mostly mayonnaise, ketchup, mustard, and sour cream-based dressings.

The school meal guidelines do not have specific recommendations for dairy products other than milk (8), whereas the Norwegian FBDG recommend choosing dairy products low in fat, salt, and sugar (21). Dairy products (other than milk) in disagreement that are categorized as products *recommended to be offered* but obtaining Nutri-Score D or E (*n* = 25, 6%) were cheese products with >10% saturated fat, whereas products categorized as *recommended to be offered in limited amounts* or *recommended not to be offered* and obtaining Nutri-Score A or B (*n* = 11, 3%) were yoghurts with low sugar and saturated fat content (Table 1).

According to the school meal guidelines, cooking oils, liquid and soft margarine should be used instead of hard margarine and butter (8). Fats and oils in disagreement categorized as products *recommended to be offered* but obtaining Nutri-Score D or E (*n* = 22, 17%) were hard margarine (misclassified by Matvalget due to manual typing errors), whereas products *recommended to be offered in limited amounts* or *recommended not to be offered* and obtaining Nutri-Score A or B were canola oils for frying (*n* = 4, 3%; Table 1). Products with Nutri-Score A, B, or C were cooking oils and soft margarine.

According to the school meal guidelines, snack products like chocolate, candy, and chips should not be offered (8), and 80% of these products received Nutri-Score D or E. Snacks in disagreement were protein puddings and low-fat ice cream, obtaining Nutri-Score A or B but classified as *recommended not to be offered* (*n* = 8, 2%; Table 1).

Overall, the school meal guidelines state that beverages should have no more than a low content of added sugars or sweeteners and fat (8). Most beverages in disagreement categorized as products *recommended to be offered* but obtaining Nutri-Score D or E (*n* = 17, 4%; Table 1) were smoothies, whereas products *recommended to be offered in limited amounts* or *recommended not to be offered* and obtaining Nutri-Score A or B (*n* = 22, 5%) were plain milk with 1% fat or flavored milk with low-fat and <5% sugars. Juice, sodas, iced coffee, and energy drinks with non-nutritive sweeteners mostly clustered in Nutri-Score C and were classified as products *recommended not to be offered* according to the guidelines, except for juice in containers ≤250 ml.

### Relevant Nutri-Score ranges for guiding food procurements

Across food groups, foods and beverages with a Nutri-Score of A or B largely aligned with the school meal guidelines. However, in some food categories, foods with C or D were also categorized as *recommended to be offered*. In summary, the following Nutri-Score ranges provide the best agreement with the guidelines:

- Overall, Nutri-Score A and B are recommended but opt for whole grain instead of refined products of flour, rice, and pasta.
- Nutri-Score C is acceptable for bread toppings and for juice in portions not exceeding 250 ml.
- Nutri-Score D is acceptable for fish-based bread toppings and ingredients used in small quantities (e.g. dressings, reduced fat cream, and mayonnaise).
- Nutri-Score E should be avoided with one exception for oil-based salad dressings.

A more detailed overview of the proposed Nutri-Score ranges based on, and in amendment to, the school meal guidelines is provided in Supplementary Table 7.

### Goals for food purchases in high schools based on Nutri-Score distribution in real-life settings

**Fig. 2:**
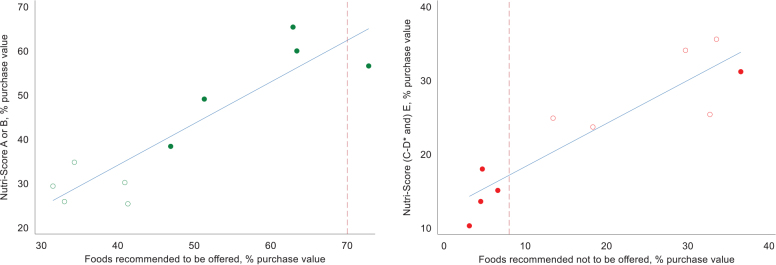
Agreement between the Nutri-Score and the school meal guidelines based on the purchase value on products being categorized as *recommended to be offered* and Nutri-Score A or B (left), and similarly, purchase value on products being categorized as *recommended not to be offered* and Nutri-Score E (*C-E for beverages) (right). Solid circle markers indicate schools that have received guidance from Matvalget. The blue line represents the estimated association. The red, dotted lines represent the goals set by Viken County for a minimum of 70% of purchases on recommended foods (left) and maximum 8% of purchases on non-recommended (right).

There was large variation between the schools in the proportion of money spent on foods and beverages classified as *recommended to be offered*, according to the school meal guidelines (32–73% of the total value, Fig. 2). This proportion was linearly associated with money spent on products classified with Nutri-Score A or B (β = 0.94, *P* < 0.001). Similarly, there was a large variation in proportion of money spent on foods and beverages classified as *recommended not to be offered*, according to the school meal guidelines (3–37% of the total value), and the proportion was significantly associated with money spent on products classified with Nutri-Score E (including C to E for beverages) (β = 0.59, *P* < 0.001). Hence, the Viken County goal of ≥70% of money spent on recommended foods and ≤8% of money spent on non-recommended foods matches to a goal of ≥65% on Nutri-Score A or B and ≤15% on E (including C to E for beverages).

## Discussion

This study indicates that the Nutri-Score has the potential of being a valuable tool for guiding and evaluating food procurements in Norwegian high schools. There was an overall good agreement between the Nutri-Score and the Matvalget’s classification tool as a proxy for the Norwegian Directorate of Health’s school meal guidelines. We present a suggestion for how Nutri-Score can be implemented in the relevant current school meal guidelines to make it clearer and easier what specific foods to offer and not to offer. Overall, Nutri-Score A and B can be recommended, but for some food groups, such as bread toppings (e.g. fish and cheese), Nutri-Score C and D can also be recommended. We also propose that a realistic goal for evaluating food procurements could be minimum 65% of the total value spent on products with Nutri-Score A or B and maximum 15% on products with Nutri-Score E (C-E for beverages). This study offers a comprehensive understanding of how Nutri-Score can serve as both a guiding tool for procurers to select healthier foods and for monitoring and evaluating the nutritional quality of food procurement in high schools. To our knowledge, this is the first study to assess the Nutri-Score 2023 version’s ability to measure school food procurements’ accordance with school meal guidelines.

Evaluating individual food items based on current school meal guidelines presents a significant challenge. For many foods or food groups, specific recommendations are not provided, necessitating the purchaser’s discernment to determine, for instance, whether the fat content in a sour cream is acceptable. This process demands extensive knowledge of food products as well as a deep understanding of dietary advice and guidelines. Matvalget’s classification tool has been found helpful to assess food procurement in Viken County and has been recommended for monitoring public food environments in Norway (22). This is also indicated in the current study that schools that receive counseling provided by Matvalget had spent a large proportion of money on food items that were classified as *recommended to be offered*. This highlights the need and impact of an additional guiding tool for public health procurers. A strength of the categorization by Matvalget is that the potential use and portion size of the food are sometimes taken into consideration so that, for example, a salad dressing is categorized as recommended regardless of nutritional quality, and a frying fat is categorized as non-recommended regardless of fat quality. The classification tool developed by Matvalget, however, is time- and resource-consuming to develop and maintain, as it relies on manual categorization and might therefore not be optimal for providing guidance or evaluation on a national level. Additionally, there have been instances where foods were misclassified by Matvalget due to manual typing errors. Nutri-Score is a consistent, objective system that could provide clear guidance and evaluation possibilities when used in addition to the school meal guidelines and thereby complement the knowledge of the procurer. However, neither the portion size nor what could be considered part of a healthy meal is considered by the score, which is a limitation.

It is likely effective to employ an algorithm-based categorization system as a tool for guiding the food procurement process. However, emphasizing product and nutrient content that necessitates algorithms to determine what to purchase and prepare might not be optimal in a broader context. There is a risk that the focus potentially shifts toward purchasing the ‘correct’ products rather than relying on one’s culinary expertise to prepare well-balanced meals. From a societal perspective, it would be ideal if chefs working in cafeterias (and preferably the general populace) could assemble varied and health-promoting meals using their food knowledge, and that it is feasible to adhere to dietary guidelines and recommendations accordingly. After all, all food procurement for cafeterias does not always go directly to the cafeterias for sale; some products or ingredients are utilized in the preparation of foods and dishes. Optimal guidance in this context should emphasize building competence and trust in the chefs’ ability to create healthy meals based on their own assessments, rather than merely understanding that a few Nutri-Score E ratings can be acceptable. This issue presents a significant dilemma and highlights the delicate nature of the associated communication.

There is potential for foods with poor nutritional quality to fit within a healthy diet, for instance, as ingredients in a healthful meal (such as salad dressing) or to increase fish intake (such as smoked salmon). It is, however, crucial to ensure effective communication so that purchasers understand that there is room for including also Nutri-Score E ratings as ingredients in an otherwise healthy meal. We propose that the maximum purchase value for food items with Nutri-Score E (C-E for beverages) could be ≤15%. The goal set by Viken County was maximum 8% of money spent on food items classified as *recommended not to be offered.* The main difference between what is included using these Nutri-Score ranges compared with using the classification *recommended not to be offered* is that the proposed Nutri-Score ranges include all drinks with non-nutritive sweeteners (e.g. iced coffee and carbonated drinks), butter and full fat cream, and a lot of processed red meat products, which were classified as *recommended to be offered in limited amounts* by Matvalget. We chose to include beverages with Nutri-Score C-E as products to limit since most juice-based drinks, sodas, iced coffee, and energy drinks with non-nutritive sweeteners, which are frequently consumed by high school students in Norway (23), clustered in Nutri-Score C.

In 2021, a validated questionnaire was published for estimating adherence to the school meal guidelines (13). The newly developed questionnaires offer an effective method for schools to monitor adherence to each specific recommendation in the school meal guidelines; however, they do not provide a comprehensive assessment of all foods offered to students and cannot be used as a guiding tool for the procurer at the moment of purchase.

The potential of Nutri-Score to aid school procurers in selecting healthier foods has yet to be investigated. A somewhat similar purchasing situation where it has been investigated if Nutri-Score can assist in selecting healthier foods is online web-portals for food procurements. Two studies from a study population consisting of students, low-income individuals, and individuals with cardiometabolic diseases found that Nutri-Score assisted in making the purchaser choose not only more unprocessed products (24) and an overall lower content of energy, saturated fat, sugars, and sodium but also lower content of protein, compared with no label in an online purchasing situation (25). Another study investigated the effect of Nutri-Score on purchase of foods with low nutritional quality and found only small positive effects (26). Further research is needed to fully decipher the intended and unintended consequences of using the Nutri-Score as a guiding tool for school food procurers.

Since the Nutri-Score is not an applied nutrient profiling system in Norway, its current usability as a guiding tool for food procurers is limited. However, the algorithms for calculating the Nutri-Score are publicly accessible and can be utilized, provided the nutritional content of the food products is available. Theoretically, the scores could be made available in the webstores, but the practicalities required for such an incorporation would need to be investigated. Even if it is not currently available for the procurer, it could still serve as a evaluation tool for assessing the overall food procurement for schools. However, it would likely be very time-consuming for the food procurer to manually calculate the Nutri-Score of all products. In contrast to the Keyhole label (27), which is a label for healthier foods adopted in Norway and several other Nordic countries, the Nutri-Score implies both healthier and less healthy food options. This allows for the evaluation of both foods that should be offered and foods that should be limited or not offered, regardless of being an implemented system or not.

### Strengths and limitations

This study was conducted using real-life data covering all purchased food items from high schools in Viken County, which covers 23% of the total population in Norway. A wide variety of foods from different brands were included, contributing to a strong representation of the foods and beverages typically procured in high school canteens. Although data from some suppliers were excluded from the analyses, comparable products from suppliers or producers were available from the three suppliers considered. Since the procurement list encompassed all purchases from one quarter of a year, it is likely that most of the items typically bought were included. Some limitations should be mentioned. We assessed the agreement with the Matvalget’s classification tool as a proxy for the Norwegian school meal guidelines. Although using this proxy means that we have not assessed the agreement with the actual guidelines, it is the only proxy available and a tool that is currently used by high schools in five counties in Norway, covering approximately 37% of the total population in Norway. In addition, the proxy variable is more detailed than the school meal guidelines, and we judge the variable to have good specificity, since it has been classified by nutrition experts. Furthermore, all defined disagreements were explored. While Viken County is currently using the proportion of money spent as a metric for monitoring adherence to the school meal guidelines, we recognize its limitations as price fluctuations in food items can significantly impact the effectiveness of this approach. Finally, we have not investigated the effect of Nutri-Score on actual food purchases but merely the potential of it.

## Conclusion

This study suggests that Nutri-Score has the potential of being an effective tool for guiding toward healthier food options and for monitoring food purchases in alignment with the Norwegian Directorate of Health’s school meal guidelines. The Nutri-Score can complement the school meal guidelines and enhance the clarity of the recommendations of food procurement in Norwegian high schools. However, since the Nutri-Score is not an applied nutrient profiling system in Norway, its current usability is limited. Furthermore, the reported low awareness of and adherence to the school meal guidelines calls for dedicated improvement measures.

## Supplementary Material



## Declarations

### Availability of data and materials

The datasets used and/or analyzed during the current study are available from the corresponding author upon reasonable request.
