# Supplementary material for: The performance of Nutri-Score as a tool in guiding and evaluating nutritional quality of food procurements for high schools in Norway
Source: Food Nutr Res. 2025 Oct 23;69:11483. doi: 10.29219/fnr.v69.11483 (PMC12604552; doi:10.29219/fnr.v69.11483)
Supplement: Supplementary file 1 [file FNR-69-11483-s1.docx]

# Supplementary material

| Table S1**.** Matvalget’s interpretation of the guidelines for food procurements in high schools | | | |  |
| --- | --- | --- | --- | --- |
|  | **Recommended to be offered** | **Recommended to be offered in limited amounts** | **Recommended not to be offered** | **Associated recommendations for food procurements in high schools** |
| **Foods** |  |  |  |  |
| **Bread and grain products** | Products >50% whole grain | Products <50% whole grain | White bread | Use whole-grain bread and grain products high in fibre and low in fat, sugar, and salt. |
| **Bread toppings** | Lean meat and cheese ≤17% fat, fish spreads | Processed red meat | Chocolate and similar | The selection of bread spread should be varied and always include fish and vegetables. |
| **Meat and fish** | Pure white meat  Fish and fish products | Pure red meat and processed red and white meat |  | When serving hot meals, there should be a variety of fish, meat, and vegetarian dishes. Red meat and processed products should be limited to two hot meals a week. |
| **Ready meals** | Containing fruits/vegetables/berries, whole grains, and a source of protein. Low in salt, sugar, and fat | Products high in salt and/or sugars, saturated fat, and low in fibre, like simple snack meals. | With poor nutritional quality, e.g., toasts with white bread, instant noodles, and similar | Foods with low salt content should be prioritized, and use of salt in cooking and on food should be limited. |
| **Dairy products** | Lean products low in sugars, milk, yogurt, and cottage cheese ≤4% fat, and without added sugars, cheese ≤17% fat. | Milk > 4% fat, cheese >17% fat, sour cream, crème fraiche, cooking and whipping cream |  |  |
| **Butter, margarine, and oil** | Oil, liquid or soft margarine | Dairy butter | Frying oil^2^ | Cooking oils, liquid, and soft margarine should be used instead of hard margarine and butter. |
| **Sweet pastries, chips, sweets, dessert products** |  |  | All products | Pastries and other products high in sugar and/or fat should be reserved for special occasions. Chocolate, candy, chips, and other snacks should not be offered. |
| **Beverages** |  |  |  |  |
| **Water** | All pure water, carbonated water, and flavoured water |  | With added sugar | Cold water should always be available, as a thirst quencher and for meals. |
| **White and flavoured milk** | ≤ 1.5 g added sugar and ≤ 0.7% fat can have added sweeteners | Added sugar ≤ 1.7% but fat content >0.7% | Added sugar > 1.7%, and  > 0.7% fat | Low-fat milk with 0.7% fat or less, regular low-fat milk (1%), or skimmed milk should be offered daily. |
| **Plant-based drinks from rice, oats, soya, and almond** | With added calcium, vitamin D, and vitamin B12. Otherwise, the same restrictions as flavoured milk | Added sugar ≤1.7% but fat content >0.7% | Added sugar >1.7%, and  >0.7% fat |  |
| **Juice and smoothies^1^** | In containers up to 250 ml or bag-in-box | In containers between 250 ml  and bag-in-box | With added sugar | If juice is offered, the serving sizes should not exceed 250 ml. Juice with added sugar or non-nutritive sweeteners should not be offered. |
| **Coffee and tea** | All coffee and tea |  | With added sugar |  |
| **Sodas and energy drinks** |  |  | All products | Soda and other beverages with added sugar or sweeteners should not be offered. |
| ^1^ Including juice-based drinks with or without carbonation. ^2^ With the assumption that the area of use is for deep-frying. | | | | |

| **Table S2. Nutri-Score main algorithm for general foods** | | | | | | | |
| --- | --- | --- | --- | --- | --- | --- | --- |
| Points | Unfavorable components | | | | Favorable components | | |
|  | Energy  (kJ per 100g) | Sugars (g per 100g) | Saturated fat (g per 100g) | Salt  (g per 100g) | Protein*  (g per 100g) | Fiber  (g per 100g) | Fruit, vegetables, legumes (%) |
| 0 | ≤ 335 | ≤ 3.4 | ≤ 1.0 | ≤ 0.2 | ≤ 2.4 | ≤ 3.0 | ≤ 40 |
| 1 | > 335 | > 3.4 | > 1.0 | > 0.2 | > 2.4 | > 3.0 | > 40 |
| 2 | > 670 | > 6.8 | > 2.0 | > 0.4 | > 4.8 | > 4.1 | > 60 |
| 3 | > 1005 | > 10 | > 3.0 | > 0.6 | > 7.2 | > 5.2 | - |
| 4 | > 1340 | > 14 | > 4.0 | > 0.8 | > 9.6 | > 6.3 | - |
| 5 | > 1675 | > 17 | > 5.0 | > 1.0 | > 12 | > 7.4 | > 80 |
| 6 | > 2010 | > 20 | > 6.0 | > 1.2 | > 14 |  |  |
| 7 | > 2345 | > 24 | > 7.0 | > 1.4 | > 17 |  |  |
| 8 | > 2680 | > 27 | > 8.0 | > 1.6 |  |  |  |
| 9 | > 3015 | > 31 | > 9.0 | > 1.8 |  |  |  |
| 10 | > 3350 | > 34 | > 10 | > 2.0 |  |  |  |
| 11 |  | > 37 |  | > 2.2 |  |  |  |
| 12 |  | > 41 |  | > 2.4 |  |  |  |
| 13 |  | > 44 |  | > 2.6 |  |  |  |
| 14 |  | > 48 |  | > 2.8 |  |  |  |
| 15 |  | > 51 |  | > 3.0 |  |  |  |
| 16 |  |  |  | > 3.2 |  |  |  |
| 17 |  |  |  | > 3.4 |  |  |  |
| 18 |  |  |  | > 3.6 |  |  |  |
| 19 |  |  |  | > 3.8 |  |  |  |
| 20 |  |  |  | > 4.0 |  |  |  |
| *Red meat products are given maximum 2 protein points. | | | | | | | |

To calculate the total score for general foods, summarize points from unfavorable components (**Table S2**).

If unfavorable component total is ≥ 11 points, then apply formula:
Nutri-Score points for general foods = total unfavorable component points - (points from the fiber component + points from the fruit, vegetables, and legumes component)

If unfavorable component total is < 11 points or the food is cheese, then apply formula:
Nutri-Score points for general foods = total unfavorable component points - total favorable component points.

Classify Nutri-Score using **Table S3**.

| **Table S3.** **Thresholds for the three Nutri-Score algorithms.** | | | | |
| --- | --- | --- | --- | --- |
| **Nutri-Score** | **Color** | **Main algorithm for general foods** | **Algorithm for fats** | **Algorithm for beverages** |
| A | Dark green | ≤0 | ≤-6 | Water |
| B | Light green | 1 to 2 | -5 to 2 | ≤2 |
| C | Yellow | 3 to 10 | 3 to 10 | 3 to 6 |
| D | Light orange | 11 to 18 | 11 to 18 | 7 to 9 |
| E | Dark orange | ≥19 | ≥19 | ≥10 |

| **Table S4. Nutri-Score total points for fats, oils, nuts, and seeds** | | | | | | | |
| --- | --- | --- | --- | --- | --- | --- | --- |
| Points | Unfavorable components | | | | Favorable components | | |
|  | Energy from saturated fat  (kJ per 100g)* | Sugars  (g per 100g) | Saturated fat/total fat (%) | Salt  (g per 100g) | Protein (g per 100g) | Fiber (g per 100g) | Fruit, vegetables, legumes (%) |
| 0 | ≤ 120 | ≤ 3.4 | < 10 | ≤ 0.2 | ≤ 2.4 | ≤ 3.0 | ≤ 40 |
| 1 | > 120 | > 3.4 | < 16 | > 0.2 | > 2.4 | > 3.0 | > 40 |
| 2 | > 240 | > 6.8 | < 22 | > 0.4 | > 4.8 | > 4.1 | > 60 |
| 3 | > 360 | > 10 | < 28 | > 0.6 | > 7.2 | > 5.2 | - |
| 4 | > 480 | > 14 | < 34 | > 0.8 | > 9.6 | > 6.3 | - |
| 5 | > 600 | > 17 | < 40 | > 1.0 | > 12 | > 7.4 | > 80 |
| 6 | > 720 | > 20 | < 46 | > 1.2 | > 14 |  |  |
| 7 | > 840 | > 24 | < 52 | > 1.4 | > 17 |  |  |
| 8 | > 960 | > 27 | < 58 | > 1.6 |  |  |  |
| 9 | > 1080 | > 31 | < 64 | > 1.8 |  |  |  |
| 10 | > 1200 | > 34 | ≥ 64 | > 2.0 |  |  |  |
| 11 |  | > 37 |  | > 2.2 |  |  |  |
| 12 |  | > 41 |  | > 2.4 |  |  |  |
| 13 |  | > 44 |  | > 2.6 |  |  |  |
| 14 |  | > 48 |  | > 2.8 |  |  |  |
| 15 |  | > 51 |  | > 3.0 |  |  |  |
| 16 |  |  |  | > 3.2 |  |  |  |
| 17 |  |  |  | > 3.4 |  |  |  |
| 18 |  |  |  | > 3.6 |  |  |  |
| 19 |  |  |  | > 3.8 |  |  |  |
| 20 |  |  |  | > 4.0 |  |  |  |
| *Energy from saturated fat = saturated fat (g per 100 grams) x 37 | | | | | | | |

To calculate the total score for fats, oils, nuts, and seeds, summarize points from unfavorable components (**Table S4**).

If unfavorable component total is ≥ 7 points, then apply formula:
Nutri-Score points for fats, oils, nuts, and seeds = total unfavorable component points - (points from the fiber component + points from the fruit, vegetables, and legumes component)

If unfavorable component total is < 7 points, then apply formula:
Nutri-Score points for fats, oils, nuts, and seeds = total unfavorable component points – total favorable component points.

Classify Nutri-Score using **Table S3**.

| **Table S5. Nutri-Score total points for beverages** | | | | | | | | |
| --- | --- | --- | --- | --- | --- | --- | --- | --- |
| Points | Unfavorable components | | | | | Favorable components | | |
|  | Energy (kJ per 100ml) | Sugars (g per 100ml) | Saturated fat (g per 100ml) | Salt  (g per 100ml) | Non-nutritive sweeteners  (absence/presence) | Protein (g per 100ml) | Fiber  (g per 100ml) | Fruit, vegetables, legumes (%) |
| 0 | ≤ 30 | ≤ 0.5 | ≤ 1.0 | ≤ 0.2 | - | ≤ 1.2 | ≤ 3.0 | ≤ 40 |
| 1 | ≤ 90 | ≤ 2.0 | > 1.0 | > 0.2 | - | > 1.2 | > 3.0 | - |
| 2 | ≤ 150 | ≤ 3.5 | > 2.0 | > 0.4 | - | > 1.5 | > 4.1 | > 40 |
| 3 | ≤ 210 | ≤ 5.0 | > 3.0 | > 0.6 | - | > 1.8 | > 5.2 | - |
| 4 | ≤ 240 | ≤ 6.0 | > 4.0 | > 0.8 | Presence | > 2.1 | > 6.3 | > 60 |
| 5 | ≤ 270 | ≤ 7.0 | > 5.0 | > 1.0 |  | > 2.4 | > 7.4 | - |
| 6 | ≤ 300 | ≤ 8.0 | > 6.0 | > 1.2 |  | > 2.7 |  | > 80 |
| 7 | ≤ 330 | ≤ 9.0 | > 7.0 | > 1.4 |  | > 3.0 |  |  |
| 8 | ≤ 360 | ≤ 10 | > 8.0 | > 1.6 |  |  |  |  |
| 9 | ≤ 390 | ≤ 11 | > 9.0 | > 1.8 |  |  |  |  |
| 10 | > 390 | > 11 | > 10 | > 2.0 |  |  |  |  |
| 11 |  |  |  | > 2.2 |  |  |  |  |
| 12 |  |  |  | > 2.4 |  |  |  |  |
| 13 |  |  |  | > 2.6 |  |  |  |  |
| 14 |  |  |  | > 2.8 |  |  |  |  |
| 15 |  |  |  | > 3.0 |  |  |  |  |
| 16 |  |  |  | > 3.2 |  |  |  |  |
| 17 |  |  |  | > 3.4 |  |  |  |  |
| 18 |  |  |  | > 3.6 |  |  |  |  |
| 19 |  |  |  | > 3.8 |  |  |  |  |
| 20 |  |  |  | > 4.0 |  |  |  |  |

To calculate the total score for beverages, summarize points from unfavorable components and favorable components separately (**Table S5**).

Nutri-Score points for beverages = total unfavorable component points – total favorable component points.

Classify Nutri-Score using **Table S3**.

**Table S6.** Mean nutritional content by Nutri-Score categories within the food groups.

|  | **N** | **Energy**  Mean ± SD | **Fat** Mean ± SD | **Saturated fat** Mean ± SD | **Sugar** Mean ± SD | **Salt** Mean ± SD | **Fiber** Mean ± SD | **Protein** Mean ± SD |
| --- | --- | --- | --- | --- | --- | --- | --- | --- |
| Fruit, vegetables, legumes | 1,335 |  |  |  |  |  |  |  |
| A-B | 1,156 | 223±245 | 0.7±2.2 | 0.1±0.4 | 4.5±3.8 | 0.1±0.2 | 2.4±2.9 | 2.3±3.7 |
| C | 65 | 609±382 | 4.1±5.7 | 0.6±0.9 | 18±21 | 1.0±0.9 | 2.7±3.1 | 2.6±5.8 |
| D-E | 114 | 925±567 | 8.4±15 | 2.3±7.1 | 30±23 | 1.8±2.5 | 1.9±3.7 | 1.7±2.9 |
| Grains | 112 |  |  |  |  |  |  |  |
| A-B | 86 | 1439±161 | 3.7±3.0 | 0.6±0.5 | 1.9±2.7 | 0.1±0.2 | 7.9±5.3 | 11±2.5 |
| C | 18 | 1150±504 | 4.1±3.7 | 1.0±0.7 | 8.1±7.3 | 0.5±0.4 | 3.4±3.7 | 5.2±3.6 |
| D-E | 8 | 1587±72 | 5.9±8.2 | 2.9±3.9 | 17±11 | 1.1±0.9 | 4.3±5.3 | 6.0±4.0 |
| Bread | 297 |  |  |  |  |  |  |  |
| A-B | 83 | 1155±218 | 4.3±2.3 | 0.7±0.4 | 2.4±1.9 | 0.9±0.3 | 8.1±5.6 | 9.0±2.0 |
| C | 167 | 1123±182 | 4.4±2.9 | 0.7±0.5 | 2.8±2.0 | 1.1±0.3 | 2.8±2.1 | 7.7±2.5 |
| D-E | 47 | 1428±358 | 12±9.0 | 3.8±4.2 | 3.4±4.2 | 1.6±0.8 | 1.9±1.5 | 8.2±3.4 |
| Pasta, rice | 111 |  |  |  |  |  |  |  |
| A-B | 99 | 1347±325 | 1.4±0.8 | 0.4±0.3 | 1.9±1.6 | 0.0±0.1 | 2.5±2.3 | 9.4±3.5 |
| C | 11 | 1167±481 | 2.3±1.9 | 0.3±0.3 | 1.2±1.6 | 0.7±0.5 | 1.5±1.4 | 5.9±4.0 |
| D-E | 1 | 1571± | 3.0± | 0.4± | 4.1± | 1.7± | 5.0± | 12± |
| Red meat | 460 |  |  |  |  |  |  |  |
| A-B | 59 | 495±95 | 3.9±2.3 | 1.4±0.9 | 0.1±0.4 | 0.1±0.1 | 0.0±0.3 | 20±4.7 |
| C | 91 | 691±191 | 9.8±5.2 | 3.7±2.3 | 0.3±0.5 | 0.8±0.7 | 0.0±0.2 | 18±3.4 |
| D-E | 310 | 1072±532 | 20±11 | 7.5±4.4 | 0.7±0.8 | 2.3±1.7 | 0.0±0.2 | 16±6.9 |
| Poultry | 152 |  |  |  |  |  |  |  |
| A-B | 79 | 642±204 | 7.8±5.5 | 2.1±1.6 | 0.2±0.4 | 0.5±0.5 | 0.0±0.1 | 20±3.2 |
| C | 26 | 589±169 | 6.7±3.8 | 1.7±0.9 | 0.6±0.6 | 1.6±0.3 | 0.1±0.3 | 16±4.5 |
| D-E | 47 | 810±211 | 13±5.9 | 3.4±1.4 | 0.9±0.9 | 1.8±0.3 | 0.0±0.0 | 14±4.4 |
| Fish, seafood | 218 |  |  |  |  |  |  |  |
| A-B | 122 | 558±257 | 5.2±5.7 | 0.8±1.0 | 0.2±0.4 | 0.5±0.5 | 0.1±0.3 | 17±4.5 |
| C | 40 | 640±263 | 8.2±6.7 | 1.3±1.1 | 1.6±1.7 | 1.4±0.3 | 0.5±0.7 | 11±1.8 |
| D-E | 56 | 977±455 | 16±13 | 2.2±1.1 | 7.7±10 | 3.5±2.3 | 0.0±0.1 | 15±7.2 |
| Ready meals | 315 |  |  |  |  |  |  |  |
| A-B | 68 | 613±350 | 4.3±3.8 | 0.7±0.7 | 2.1±1.9 | 0.6±0.4 | 1.5±2.4 | 8.3±6.7 |
| C | 161 | 616±372 | 7.6±8.6 | 1.7±1.2 | 4.1±3.5 | 0.7±0.4 | 0.6±1.2 | 4.4±3.4 |
| D-E | 86 | 1210±348 | 17±9.8 | 6.2±4.1 | 3.5±4.9 | 1.5±1.3 | 1.3±3.4 | 11±8.4 |
| Sauces, dressings | 332 |  |  |  |  |  |  |  |
| A-B | 46 | 121±143 | 0.8±2.6 | 0.1±0.3 | 2.8±3.6 | 0.5±0.3 | 1.1±2.5 | 1.4±2.6 |
| C | 73 | 431±376 | 6.0±10 | 0.8±1.4 | 7.9±8.1 | 1.0±0.5 | 0.3±0.7 | 1.8±1.7 |
| D-E | 213 | 1318±862 | 26±27 | 2.8±3.8 | 13±17 | 3.5±1.0 | 0.5±1.3 | 2.5±2.7 |
| Dairy products, eggs | 387 |  |  |  |  |  |  |  |
| A-B | 68 | 404±296 | 4.1±4.1 | 1.6±1.2 | 3.4±2.8 | 0.2±0.2 | 0.3±1.1 | 9.4±11 |
| C | 67 | 538±251 | 7.7±5.5 | 4.3±3.4 | 6.5±4.3 | 0.3±0.4 | 0.0±0.2 | 7.7±7.4 |
| D-E | 252 | 1357±322 | 27±8.1 | 17±5.0 | 4.3±10 | 1.3±0.8 | 0.0±0.1 | 16±10 |
| Fats | 131 |  |  |  |  |  |  |  |
| A-B | 42 | 2893±1399 | 75±42 | 8.3±5.6 | 0.2±0.8 | 0.0±0.1 | 0.0±0.2 | 0.3±0.9 |
| C | 30 | 2254±1137 | 56±36 | 9.2±5.3 | 1.1±4.1 | 0.5±0.5 | 1.0±5.2 | 1.3±3.6 |
| D-E | 59 | 2408±1003 | 63±29 | 37±20 | 2.4±11 | 0.6±0.6 | 0.6±2.8 | 0.9±1.4 |
| Nuts | 73 |  |  |  |  |  |  |  |
| A-B | 43 | 2347±490 | 50±10 | 5.3±2.0 | 2.8±2.9 | 0.1±0.1 | 11±9.4 | 20±4.8 |
| C | 28 | 2340±314 | 41±13 | 6.7±3.1 | 17±15 | 0.7±0.5 | 5.9±3.5 | 18±6.0 |
| D-E | 2 | 2315±143 | 32±3.8 | 7±4.1 | 17±18 | 1.1±1.3 | 5.4±1.8 | 12±0.7 |
| Snacks | 456 |  |  |  |  |  |  |  |
| A-B | 9 | 591±388 | 2.1±3.2 | 0.9±1.0 | 4.2±4.5 | 0.1±0.2 | 6.4±12 | 3.4±3.7 |
| C | 84 | 780±461 | 5.3±5.8 | 1.8±1.7 | 13±7.8 | 0.3±0.3 | 0.8±1.5 | 3.2±2.8 |
| D-E | 363 | 1671±415 | 18±10 | 8.1±6.6 | 37±22 | 0.5±0.6 | 1.5±3.0 | 5.5±4.3 |
| Beverages | 416 |  |  |  |  |  |  |  |
| A-B | 126 | 74±85 | 0.4±0.6 | 0.2±0.4 | 1.7±2.2 | 0.0±0.0 | 0.0±0.1 | 1.4±1.8 |
| C | 145 | 142±101 | 0.7±1.2 | 0.4±0.8 | 4.8±3.8 | 0.1±0.1 | 0.0±0.2 | 1.4±1.7 |
| D-E | 145 | 217±149 | 0.6±1.8 | 0.3±1.1 | 10±5.1 | 0.1±0.1 | 0.1±0.4 | 0.8±1.7 |

SD, Standard deviation

| **Table S7.** Proposed food-group specific Nutri-Score ranges to be recommended in the current Norwegian school meal guidelines. | | |
| --- | --- | --- |
| **Current school meal guideline** | **Proposed supplemental text for the guideline** | **Proposed supplemental text for the rationale for each recommendation** |
| **#6:** The food and beverage offering should be based on the Norwegian Directorate of Health's Food-Based Dietary Guidelines. | Within food groups, a more favourable Nutri-Score reflects healthier choices. | Nutri-Score, a five-label scale from A (highest nutritional quality) to E (lowest nutritional quality), will, within food groups, guide towards healthier choices. It is recommended to choose the product with the most favourable Nutri-Score within each food group. |
| **#9:** Use whole grain bread and grain products high in fibre and low in fat, sugar, and salt. | Offer whole grain bread and grain products with Nutri-Score A or B. | Grain products like rice and pasta with Nutri-Score A and B generally have a high fibre content and a low salt content. |
| **#10:** The selection of bread spread should be varied and always include fish and vegetables. | Choose bread spreads with Nutri-Score A, B, or C. The selection of fish spreads should be varied and fish spreads with Nutri-Score D or E should be offered less often than products with Nutri-Score A, B, or C. | Fish spreads high in salt typically receive a Nutri-Score of D or E. Given that most adolescents have an inadequate intake of fish, it is recommended to provide a variety of fish spreads daily. However, fish spreads with a Nutri-Score of A, B, or C should be offered more frequently than those with a Nutri-Score of D or E. Cheese receiving Nutri-Score A, B, or C should be offered rather than cheese with Nutri-Score D. |
| **#11:** When serving hot meals, there should be a variety of fish, meat, and vegetarian dishes. | Offer pure white meat and fish receiving Nutri-Score A, B, or C. Red meat and processed products should be limited to two hot meals a week | When cooking meals, most ingredients should have Nutri-Score A, B or C. Lean, unprocessed red meat and processed white meat can get Nutri-Score A and B and should preferably be offered when serving red meat or processed white meat products. |
| **#12:** Cooking oils, liquid, and soft margarine should be used instead of hard margarine and butter. | Use oils or margarine receiving Nutri-Score A, B, or C. | Oils, liquid- and soft margarines receive mostly Nutri-Score A, B, or C, while butter, hard margarine and other hard fats mostly receive Nutri-Score D or E. |
| **#14:** Cold water should always be available, as a thirst quencher and for meals  **#15:** Low-fat milk with 0.7% fat or less, regular low-fat milk, or skimmed milk should be offered daily.  **#16:** If juice is offered, the serving sizes should not exceed 250 ml.  **#17:** Sodas, cordials, and other beverages with added sugar or sweeteners should not be offered. | Beverages with Nutri-Score A or B should be offered. Water should always be available as a thirst quencher and milk should be offered daily. Juice can be offered, but the serving size should not exceed 250 ml. | Only water receives Nutri-Score A, while beverages receiving Nutri-Score B are mostly low-fat milk (≤1% fat) or flavoured milk low in sugar and fat. Juices mostly receive Nutri-Score C but can be offered in 250 ml containers. Beverages with added non-nutritive sweeteners receive Nutri-Score C or poorer and are not recommended. |
